# A systematic review considering the risk of bias in orthodontic RCTs over 55 years

**DOI:** 10.1093/ejo/cjaf083

**Published:** 2025-10-22

**Authors:** Sofia Petrén, Lars Bondemark, Mikael Sonesson, Liselotte Paulsson

**Affiliations:** Department of Orthodontics, Faculty of Odontology, Malmö University, Carl Gustavs väg 34, Malmö SE-205 06, Sweden; Department of Orthodontics, Faculty of Odontology, Malmö University, Carl Gustavs väg 34, Malmö SE-205 06, Sweden; Department of Orthodontics, Faculty of Odontology, Malmö University, Carl Gustavs väg 34, Malmö SE-205 06, Sweden; Department of Orthodontics, Faculty of Odontology, Malmö University, Carl Gustavs väg 34, Malmö SE-205 06, Sweden

**Keywords:** RCTs, orthodontics, risk of bias, systematic review

## Abstract

**Background:**

When assessing the effectiveness of interventions, randomized controlled trials (RCTs) are considered to generate the highest level of evidence. The CONSORT 2010 recommendations promote clear and transparent reporting of RCTs. However, to perform a complete analysis of methodological errors and risk of bias that RCTs may be subjected to, the Risk of Bias tool 2 (RoB 2) has been constructed.

**Objectives:**

The aim of this systematic review was to assess the last 55 years of changes in methodological quality of orthodontic RCTs by using the RoB 2 tool.

**Search methods:**

The MEDLINE via Entrez PubMed, Web of Science, and Cochrane library databases were searched for orthodontic RCT publications from 1 January 1968 to 31 December 2024.

**Selection criteria:**

All RCTs on humans in the field of orthodontics were included. Non-randomized trials, animal studies, orthognathic surgery, syndrome patients, CLP patients, sleep apnea, and *in vitro* studies were excluded.

**Data collection and analysis:**

The quality assessment of the studies was conducted using the Cochrane Risk of Bias Tool (RoB2). The RCTs were divided into three time periods, i.e. before CONSORT 2010, from 2011 to 2016, and from 2017 to 2024.

**Results:**

A total of 3135 RCTs were identified, and after excluding studies not fulfilling the criteria, 1231 RCTs were included in the quality assessment. Publications in the later or latest time-period had a larger number with low risk of bias than early ones. However, significant room for improvement remained since, in the latest time period there were relatively many RCTs with high risk of bias (67.6%). The main factors to high bias were unclear or missing information about ‘who generated the random allocation sequence and enrolled participants’ (selection bias), whether ‘ITT or intention-to-treat’ was used (attrition bias) as well as omitting reporting of ‘all important harms or unintended effects’ (other bias). Another factor was that baseline characteristics were missing (selection bias).

**Limitations:**

The RoB2 tool is complex, and it requires trained individuals to use the tool.

**Conclusions:**

There remains a need to enhance the quality of RCTs in orthodontics. To reduce the risk of bias, researchers should become well-acquainted with RoB2 before developing research protocols and plans. Specifically, use the ‘signaling questions of RoB2’ as extensively as possible to aid in designing the plans and protocols concerning the trial's conduct and progression.

**Registration:**

PROSPERO: CRD42023390206.

## Introduction

There has been a marked rise in the number of orthodontic publications in recent times. A bibliometric MEDLINE survey between 1968 and 2017 reported that the randomized controlled trials (RCTs) accounted for 0.1% of all orthodontic publications in the 1970s and 1980s, and the proportion of RCTs increased and amounted to 2.7% over the years 2008 to 2017 [1]. It is well known that in an evidence-based context, and when assessing the effectiveness of interventions, RCTs are considered to generate the highest level of evidence. One reason for this is that the random distribution of study subjects into different groups helps to avoid selection bias. Another reason is that, thanks to the random procedure, confounding factors (known or unknown variables) that may affect the study results and are difficult to control, are equally distributed between the included groups. Many also argue that RCTs have contributed to important medical development because implementation of RCT results has shaped and improved clinical practice.

The reliability or credibility of an RCT can vary considerably. In other words, an RCT with low risk of bias cannot be equated with an RCT with high risk of bias. Despite the CONSORT 2010 recommendations [2], which aimed to promote clear and transparent reporting of RCTs, the reporting and methodology of RCTs carried out in dentistry and orthodontics have been questioned and is far from ideal and require improvements [3–5]. However, not all methodological aspects are covered by the CONSORT 2010. To perform a complete analysis of methodological errors or evaluate the risk of bias that RCTs may be subjected to, an instrument, Risk of Bias tool 2 (RoB 2), has been constructed to make a further and complete classification of the bias (low to high), which is not possible with the Consort 2010 [6]. The classification is usually graded between high and low risk of bias and is originally based on five risk domains:

- risk of bias arising from the random allocation (selection bias)
- risk of bias due to deviations from the intended interventions (performance bias)
- risk of bias due to missing outcome data (attrition bias)
- deviations in measurement of outcomes (detection bias)
- risk of bias in selection of the reported result (reporting bias)

It cannot be emphasized enough that, despite all the advantages of RCTs, it is extremely important that the trials are of high quality because poorly conducted and poorly reported trials contribute to the deterioration of clinical practice as these trials may underpin health care recommendations. Thus, suboptimal methodology and highly biased studies can result in ineffective or even potentially harmful treatment interventions being advocated in patient management [5].

Against the background of the above considerations, the aim of this systematic review was to assess and evaluate the last 55 years of changes in methodological quality of orthodontic RCTs by using the RoB 2 tool. The assessment was divided in three time periods, i.e. before CONSORT 2010, from 2011 to 2016, and 2017–2024. Our hypothesis was that publications in the later or latest time-period have lower risk of bias and improved methodological quality than early ones.

## Materials and Methods

### Protocol and registration

The protocol for this review was prepared *a priori* and was registered in PROSPERO with identification number CRD42023390206. The conduct and reporting of this systematic review was based on the Cochrane Handbook [7] and the Preferred Reporting Items for Systematic reviews and Meta-Analyses (PRISMA) statement [8], respectively.

### Study selection

The MEDLINE via Entrez PubMed, Web of Science, and Cochrane library databases were searched for orthodontic RCT publications on humans from 1 January 1968 to 31 December 2024. The search was conducted with the assistance of a specialist in informatics at the Library, Faculty of Odontology, Malmö University, Sweden. Keywords used for the search were ‘orthodontics’ OR ‘orthodontic’ OR ‘orthodontically’ AND ‘randomized controlled trial’ OR ‘randomised controlled trial’ OR ‘randomized clinical trial’ OR ‘randomised clinical trial’ OR ‘RCT’.

The eligibility of studies was independently assessed by four researchers, who are also the authors of this article. The titles and abstracts of all relevant studies were reviewed. If at least one reviewer deemed a study relevant or if the title and abstract lacked sufficient information, the full-text version of the article was retrieved. Following a preset protocol and initial inclusion and exclusion criteria, the full-text articles were independently analysed and evaluated by the four researchers. In instances of disagreement, the articles were reread and discussed until a consensus was reached.

The exclusion criteria were non-randomized trials, RCTs performed on animals, orthodontic treatments in combination with orthognathic surgery, interventions on syndrome patients including cleft lip and palate patients, mandibular advancement splints for the treatment of snoring and sleep apnea, *in vitro* studies which also include studies using extracted human premolars, systematic and narrative reviews. No search restriction was applied for publication language but papers not in English were included if they could be translated into English. The reference list of retrieved articles was also searched for additional studies.

### Data extraction

All orthodontic RCTs were screened and evaluated regarding the number of RCT publications per year, publications before or after CONSORT 2010, and which scientific journal of publication. Furthermore, data extractions were performed considering the primary outcome of the trial, whether it was a single center or multicenter RCT as well as if randomization of individuals (parallel-arm design) or within individuals (split-mouth design) was performed. Moreover, a categorization of the topic investigated in each RCT was noted. Thus, the RCTs were grouped into the following: treatment studies in general, treatment studies considering accelerated tooth movement, health economics, quality of life, adverse effects, stability and retention, material studies, implementation studies, and other, i.e. everything not included in the above.

Finally, the included RCTs were divided into three groups based on when they were published, i.e. before 2011 (before CONSORT 2010), from 2011 to 2016, and from 2017 to 2024.

### Assessment of risk of bias

To assess the risk of bias (methodological errors), every RCT was judged according to the RoB 2 tool. The tool originally created by Higgins et al. [6] has been later revised by Sterne et al. [6]. The original tool is structured into five domains through which bias might be introduced into the result and the name of the domains are descriptions of the causes of bias addressed in the domain. In this evaluation, a further domain, ‘other bias’ was added, and thereby, the six domains covered most or all types of bias that can affect results of RCTs (Table 1).

**Table 1.** The six domains of bias.

Selection bias	Bias arising from the randomization process
Performance bias	Bias due to deviations from intended interventions
Attrition bias	Bias due to missing outcome data
Detection bias	Bias in measurement of the outcome
Reporting bias	Bias in selection of the reported result
Other bias	For example, too small sample size, conflicts of interest, etc

The judgment of risk of bias was determined from the answers to a series of ‘signaling questions’ about the trial's conduct and course. For each risk domain, between two and six specific signaling questions were used to the judgments about risk of bias. The three possible answers to the signaling questions were: yes, no, and no information. If the answer was ‘yes’ this implies low risk of bias while ‘no’ means high risk of bias. If ‘no information’ was present, this was usually categorized as high risk of bias.

Below is described for each risk domain what the signaling questions applied to and depending on the answer what type of bias should apply.

#### Bias arising from the randomization process (selection bias)

- if group allocation was randomized (if yes = low risk of bias; if no = high risk of bias)
- if future group affiliation could not be predicted, if it was unknown until the participants were allocated (concealed allocation sequence) (if yes = low risk of bias; if no = high risk of bias)
- if the allocation was carried out by a person who was not involved in the trial to guarantee the hidden allocation (if yes = low risk of bias; if no = high risk of bias)
- if baseline data had imbalances indicating flaws in the randomization process (if yes = high risk of bias; if no = low risk of bias)

#### Bias due to deviations from intended interventions (performance bias)

- if participants did not know which intervention they were assigned to during the trial (if yes = low risk of bias; if no = high risk of bias)
- if the therapists did not know which interventions the participants were assigned to during the trial (if yes = low risk of bias; if no = high risk of bias)
- if knowledge of the trial and the group division could lead to deviations that were unbalanced between the groups (e.g. changes in other care or deviations from clinical practice) (if yes = high risk of bias; if no = low risk of bias)
- if the imbalance likely affected the outcome (if yes = high risk of bias; if no = low risk of bias)
- if an appropriate analysis method was used to estimate the effect (if yes = low risk of bias; if no = high risk of bias)
- if the result was seriously affected by the fact that the participants were not analysed in the group they were randomized to (if yes = high risk of bias; if no = low risk of bias)

#### Bias due to missing outcome data (attrition bias)

- if results were reported for all or almost all participants (if yes = low risk of bias; if no = high risk of bias)
- if it has been shown that the results are robust despite the dropout (e.g. with sensitivity analyses) (if yes = low risk of bias; if no = high risk of bias)
- if the dropout is highly likely to be related to the outcome measure (if yes = high risk of bias; if no = low risk of bias)
- if the dropout and the reasons for the dropout were similar between the groups (if yes = low risk of bias; if no = high risk of bias)
- if intention-to-treat (ITT) was used, i.e. all participating subjects were analysed, even those who started the treatment but did not complete it (if yes = low risk of bias; if no = high risk of bias)
- if per protocol analysis was used, i.e. only those who fulfilled the trial was analysed (if yes = high risk of bias; if no = low risk of bias)

#### Bias in measurement of the outcome (detection bias)

- if data collection did not differ between groups (if yes = low risk of bias; if no = high risk of bias)
- if those who measured the outcome were not aware of which intervention the participants received, and that the assessment was not most likely influenced by this (if yes = low risk of bias; if no = high risk of bias)

#### Bias in selection of the reported result (reporting bias)

- if the analyses were conducted according to a plan published before outcome data were available or if the trial before initiation has been registered in a database as ClinicalTrials.gov PRS (Protocol Registration and Results System) (if yes = low risk of bias; if no = high risk of bias)
- if the reported outcomes have been selected from multiple ways of measuring the outcome (e.g. different scales, time points) (if yes = low risk of bias; if no = high risk of bias)
- if the reported results have been selected from different analyses of the same outcome (if yes = low risk of bias; if no = high risk of bias)

#### Other bias

- if the authors declare that they have financial interests that could influence the outcome (if yes = high risk of bias; if no = low risk of bias)
- if the authors declare that they have other ties that could affect the outcome (if yes = high risk of bias; if no = low risk of bias)
- if the trial consists of a sufficient number of subjects and especially if a sample size calculation has been performed (if yes = low risk of bias; if no = high risk of bias)
- if those who assessed the clinical registrations are calibrated or that intra- or inter-individual comparisons have been performed (if yes = low risk of bias; if no = high risk of bias)

### Total risk of bias

Each risk domain can be assessed as having low risk of bias, moderate or some concerns about risk of bias, and high risk of bias.

#### Judgement of total risk of bias

- for an RCT to be classified with a total low risk of bias, the trial is judged to be at low risk of bias for all domains
- for an RCT with some concerns, the trial is judged to raise some concerns in at least one domain, but not to be at high risk of bias for any domain
- for high risk of bias, the trial is judged to be at high risk of bias in at least one domain or the trial is judged to have some concerns for multiple domains in a way that substantially lowers confidence in the result.

### Reliability

The titles and abstracts of all retrieved articles were scrutinized for relevance and independently by the four reviewers. To evaluate the agreement in assessments of risk of bias as well as for the above data extractions between the four reviewers, 20 randomly selected RCTs were used. Each RCT contained 50 possible items, and thereby, the 20 RCTs corresponded to a total of 1000 items to decide on which were independently rated and all were blinded to each other's results. Sixty-seven discrepancies were identified, and these were successfully resolved by consensus. Thus, the error rate was 6.7%. Cohen’s Kappa score [9] was used to measure the level of intra- and inter-examiner agreement, which corresponded to an agreement of at least 0.88, i.e. very good agreement [10]. Since the inter-examiner agreement was very good, the strategy was to divide the retrieved RCTs into four groups. Then, the four reviewers were assigned a group of RCTs each to assess.

## Results

**Figure 1.**
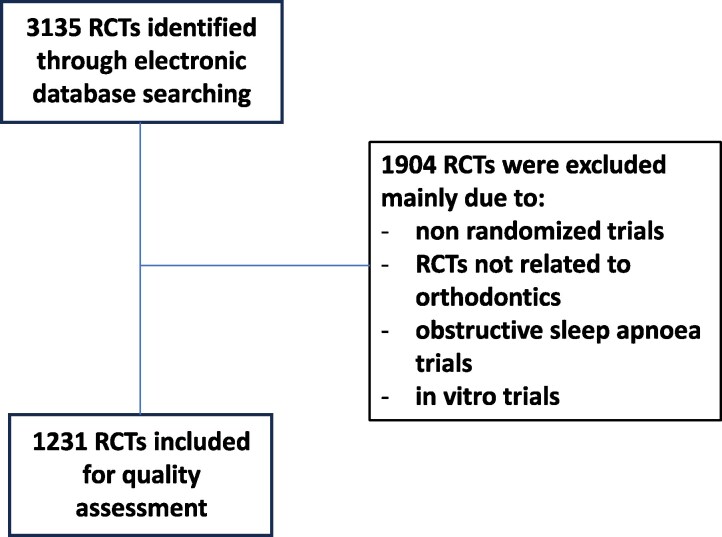
Flow chart of the included and excluded studies.

A total of 3135 RCTs were identified by the electronic search, and the main reasons for exclusions are shown in Fig. 1. Thus, for the quality assessment 1231 RCTs were included. There was a general increase in the number of RCTs over time. Thus, 206 RCTs were published before 2011, 303 from 2011 to 2016, and 722 RCTs from 2017 to 2024.

Before 2011, i.e. before CONSORT 2010, there were very few RCTs (0.5%) with low bias, (Table 2), i.e. almost all RCTs had high bias (98.5%). The later the RCTs were published, significantly more RCTs had low bias, i.e. from 2011 to 2016 the proportion was 5%, and from 2017 to 2024 low bias was found in 23.4% of the RCTs (Table 2). However, even from 2017 to 2024, there were still relatively many RCTs with high bias (67.6%), (Table 2).

**Table 2.** Total risk of bias based on the risk of bias tool 2 (RoB 2) for articles published before 2011 (A), 2011–2016 (B), and 2017–2024 (C).

Total risk of bias	Publication year						
	A: Before 2011 Total N = 206		B: 2011 to 2016 Total N = 303		C: 2017 to 2024 Total N = 722		
	N	%	N	%	N	%	*P*-value^a^
Low	1	0.5	15	5.0	169	23.4	A/B < 0.001 A/C < 0.001 B/C < 0.001
Moderate	2	1.0	20	6.6	65	9.0	
High	203	98.5	268	88.4	488	67.6	

Statistical significance *P* < 0.05.

^a^Fisher´s Exact test.

Regarding the reporting of selected items from the CONSORT 2010 checklist, presented in Table 3, the reporting of these items increased significantly the later the RCT was published. The exception was that the item ‘number of participants per group’ which was almost always reported regardless of whether in early or late publications, (Table 3). For the group of late published RCTs, the reporting of items was relatively good except for ‘who generated the random allocation sequence and enrolled participants’ (selection bias), ‘intention-to-treat (ITT)’ (attrition bias) and ‘all important harms or unintended effects’ (other bias). These three items were reported in 50.6, 31.3, and 40.4% of the RCTs (Table 3).

**Table 3.** Reporting of selected items from CONSORT checklist 2010 for RCTs published before 2011 (A), 2011–2016 (B), and 2017–2024 (C).

Checklist item	Publication year						
Reported in the RCT/article	A: Before 2011 N = 206		B: 2011 to 2016 N = 303		C: 2017 to 2024 N = 722		
	N	%	N	%	N	%	*P*-value^a^
Identification as a randomized trial in the title	88	42.7	177	58.4	588	81.4	A/B < 0.001 A/C < 0.001 B/C < 0.001
Number of participants per group	199	96.6	296	97.7	711	98.5	A/B NS A/C NS BC NS
For each group, losses and exclusions after randomization	101	49.0	186	61.4	604	83.7	A/B 0.06 A/C < 0.001 B/C < 0.001
Completely defined pre-specified primary and secondary outcome measures, including how and when they were assessed	129	62.6	232	76.6	658	91.1	A/B < 0.001 A/C < 0.001 B/C < 0.001
Sample size related to primary outcome	85	41.3	164	54.1	541	75.0	A/B 0.05 A/C < 0.001 B/C < 0.001
Mechanism used to implement the random allocation sequence	80	38.8	151	50.0	543	75.2	A/B 0.014 A/C < 0.001 B/C < 0.001
Who generated the random allocation sequence and enrolled participants	35	17.0	93	30.7	365	50.6	A/B < 0.001 A/C < 0.001 B/C < 0.001
Intention-to-treat (ITT)	35	17.0	82	27.1	226	31.3	A/B 0.08 A/C < 0.001 B/C NS
Table showing baseline characteristics	60	29.1	116	38.3	447	61.9	A/B 0.037 A/C < 0.001 B/C < 0.001
All important harms or unintended effects	7	3.4	45	14.9	292	40.4	A/B < 0.001 A/C < 0.001 B/C < 0.001
No blinding	112	54.4	115	38.1	165	22.9	A/B < 0.001 A/C < 0.001 B/C < 0.001

Statistical significance *P* < 0.05.

^a^Fisher’s Exact test

The majority of the included RCTs were single-center (72.9%), followed by split-mouth (17.7%) and multi-center trials (9.4%). When comparing the trial design in relation to the risk of bias, it was found that multi-center trials accounted for 25.0% of low-risk bias, single-center for 15.4%, and split-mouth trials for 8.3%. Thus, there were significantly more multi-center trials with low bias compared to single-center and split-mouth trials. Furthermore, there were significantly more single-center trials with low bias compared to split-mouth trials.

Table 4 presents the distribution of topics among the RCTs. The main topic was studies about adverse effects (32.7%) followed by treatment studies in general (30.3%), and treatment studies considering accelerated tooth movement (11.0%). Material studies (6.9%) and stability and retention (6.8%) were relatively common. Few RCTs were related to health economics (1.1%), implementation studies (1.1%) or quality of life (0.9%). As for topics other than above (9.3%), these mainly considered toothbrushing techniques for cleaning fixed appliances or the impact of different types of mouthwashes on plaque and gingivitis in orthodontic patients.

**Table 4.** Distribution of topics among the included RCTs, both overall and presented by time period.

Topic	Publication year						Total	
	Before 2011 N = 206		2011 to 2016 N = 303		2017 to 2024 N = 722		N = 1231	
	N	%	N	%	N	%	N	%
Treatment studies in general	82	39.8	95	31.4	196	27.1	373	30.3
Treatment studies considering accelerated tooth movement	4	1.9	21	6.9	110	15.2	135	11.0
Health economics	1	0.5	3	1.0	10	1.4	14	1.1
Quality of life	2	1.0	0	0.0	9	1.2	11	0.9
Adverse effects	58	28.2	120	39.6	224	31.0	402	32.7
Stability and retention	11	5.3	15	5.0	58	8.0	84	6.8
Studies on orthodontic materials	24	11.7	23	7.6	38	5.3	85	6.9
Implementation studies	3	1.5	1	0.3	9	1.2	13	1.1
Other, i.e. everything not included in the above.	21	10.2	25	8.3	68	9.4	114	9.3

During the past 55 years, the RCTs have been published in more than 61 different scientific journals. Table 5 presents 11 journals that had the highest number and at least 25 published RCTs during the 55-year period. These 11 journals contributed to 70% of all the RCTs while the five journals American Journal of Orthodontics and Dentofacial Orthopedics, the Angle Orthodontist, European Journal of Orthodontics, Journal of Orofacial Orthopedics and Journal of Orthodontics accounted for 50.4% of all the published RCTs. Of the non-orthodontic journals, Clinical Oral Investigation and BMC Oral Health published the greatest number of orthodontic RCTs (Table 5).

**Table 5.** Orthodontic RCT publications in 11 journals that had the highest number of RCTs from 1968 to 2024.

Journal (abbreviated)	N	Percent	Cumulative percent	JIF 2023
Am J Orthod Dentofacial Orthop	207	16.8	16.8	2.7
Angle Orthod	172	14.0	30.8	3.0
Eur J Orthod	170	13.8	44.6	2.8
J Orthod	50	4.1	48.7	1.4
J Orofac Orthop	49	4.0	52.7	1.3
Clin Oral Invest	45	3.7	56.4	3.1
BMC Oral Health	38	3.1	59.5	2.6
Orthod Craniofac Res	37	3.0	62.5	2.4
Int Orthod	37	3.0	65.5	1.8
Prog Orthod	31	2.5	68.0	3.5
Dent Press J Orthod	29	2.4	70.4	1.1

Also, the journal impact factor (JIF) 2023 is given.

Table 5 also presents the impact factor of the 11 journals in 2023. In general, the impact factors are relatively low with the highest at 3.5, i.e. Progress in Orthodontics while the Dental Press Journal of Orthodontics has the lowest at 1.1. It can also be noted that the Angle Orthodontist, Dental Press Journal of Orthodontics, Progress in Orthodontics and BMC Oral Health are open access journals.

In Table 6, the journals’ proportion of RCTs with low, moderate, and high bias is presented. Before 2011 or before CONSORT 2010, the journals published very few or no RCTs with low bias. For all journals, the later the RCTs were published, the greater the proportion of RCTs with low bias (Table 6). European Journal of Orthodontics was the journal that during the whole 55-year period published the highest amount RCTs with low bias (30.6%), followed by International Orthodontics (27.0%) and Progress in Orthodontics (25.8%). If we consider the most recent period, i.e. from 2017 to 2024, during which most RCTs were published with low bias, Journal of Orthodontics published 52.9% RCTs with low bias. The result of European Journal of Orthodontic was 44.4%, Progress in Orthodontics had 40.0% and for Orthodontics and Craniofacial Research the proportion with low bias was 35% (Table 6).

**Table 6.** The journals’ proportion of RCTs with low, moderate, and high bias is presented during the whole 55-year period as well as the proportions of low bias before 2011, 2011 to 2016, and 2017 to 2024.

Journal	Bias for the entire 55-year period							Proportion low bias					
		Low		Moderate		High		Before 2011		2011 to 2016		2017 to 2024	
	Total N	N	%	N	%	N	%	N	%	N	%	N	%
Am J Orthod Dentofacial Orthop	207	21	10.1	17	8.2	169	81.6	1	1.2	7	11.9	13	20.3
Angle Orthod	172	16	9.3	10	5.8	146	84.9	0	0.0	2	3.7	14	15.2
Eur J Orthod	170	52	30.6	24	14.1	94	55.3	0	0.0	4	11.1	48	44.4
J Orthod	50	10	20.0	3	6.0	37	74.0	0	0.0	1	6.7	9	52.9
J Orofac Orthop	49	6	12.2	0	0.0	43	87.8	0	0.0	0	0.0	6	17.1
Clin Oral Invest	45	10	22.2	2	4.4	33	73.3	0	0.0	0	0.0	10	27.8
BMC Oral Health	38	8	21.1	4	10.5	36	68.4	0	0.0	0	0.0	8	22.2
Orthod Craniofac Res	37	7	18.9	3	8.1	27	73.0	0	0.0	0	0.0	7	35.0
Int Orthod	37	10	27.0	5	13.5	22	59.5	0	0.0	0	0.0	10	29.4
Prog Orthod	31	8	25.8	2	6.5	21	67.7	0	0.0	0	0.0	8	40.0
Dent Press J Orthod	29	4	13.8	1	3.4	24	82.8	0	0.0	0	0.0	4	19.0

## Discussion

The aim of this systematic review was to assess and evaluate the last 55 years of changes in methodological quality of orthodontic RCTs by using the RoB 2 tool. Our hypothesis that publications in the later or latest time-period have lower risk of bias and improved methodological quality than early ones was clearly verified.

The enhancement over time regarding low risk of bias was not surprising. Over the years this quality strengthening in orthodontic research has been well clarified in the research context. However, there was still great room for improvement as the trials with high risk of bias still constitute 67.6%.

The main factors that led to quality deficiencies or high bias were unclear or missing information about ‘who generated the random allocation sequence and enrolled participants’ (selection bias), whether ‘ITT or intention-to-treat’ was used (attrition bias) as well as omitting reporting of ‘all important harms or unintended effects’ (other bias). Another factor was that baseline characteristics were missing (selection bias).

The accuracy of study reporting has a major impact and connection to the results. Of course, well-reported studies usually have a lower risk of bias through accurate and thorough reporting. An obvious factor in this context is the CONSORT 2010 recommendations [2]. These aims are to promote clear and transparent reporting of RCTs. Therefore, many RCTs with moderate or high RoB were partly due to inadequate reporting.

The novelty of this systematic quality review was the use of the RoB2 tool. It is well known that RoB2 is recommended for assessing the risk of bias in randomized trials. RoB2 reflects the understanding of how causes of bias can affect study results, and therefore, RoB2 has been a very suitable tool for assessing and evaluating bias. However, RoB2 is a detailed and comprehensive tool that can be difficult and demanding, even for raters with significant expertise in systematic reviews. To improve reliability, calibration exercises and intensive training are required. It is also important to be aware that some variables may be very crucial to the final assessment, whereby a change from low to moderate or from moderate to high RoB assessment will be the result. Consequently, even if only one variable gave a ‘no’, the risk of bias could go from low to moderate and from moderate to high.

Previous analyses of the quality of orthodontic RCTs have also been conducted, but in these the reporting quality has been assessed based on the Consort 2010 guidelines [4, 11, 12]. As with our review, they found that the reporting quality of RCTs, including split-mouth trials, has improved the later the RCTs were published. Furthermore, these evaluations concluded, just like ours, that there is much room for improvement and that the overall quality was still suboptimal.

It is not only in orthodontics that the quality of RCTs has been assessed. The study design and risk of bias related to the primary outcome in RCTs in periodontics have been evaluated between 2018 and 2020 [13]. It was found that improvement in the quality of RCTs in periodontics is still needed. Consequently, 39% of the RCTs had a high risk, and 23% had a low risk of bias. These results are quite consistent with our assessment conducted from 2017 to 2024.

It has been argued that publications in high-impact journals may have a higher proportion of low bias compared to articles published in lower-impact journals. Conceivably, this could be explained by a more rigorous review process of publications carried out in journals with higher impact factors. However, there is a great deal of uncertainty with such an assumption, and in fact, the journal impact factor has been considered a poor measure of research quality [14]. Regardless of whether journal impact factor may play a role, the vast majority of orthodontic RCTs were published in journals with low impact factors, i.e. impact factors for year 2023 between 1.1 and 3.5. The journals with the highest proportion of publications with low bias were in European Journal of Orthodontics, Progress in Orthodontics, Journal of Orthodontics, Orthodontics and Craniofacial Research and International Orthodontics.

Most of the topics among the RCTs concerned adverse effects and treatment studies in generally. The studies about adverse effects included for example studies about root resorption, white spot lesions and pain. The distribution of topics remained quite stable during the different time periods, except for studies on increased tooth acceleration, which increased from 1.9% to 15.2%. Malocclusions and orthodontic treatment's impact on Oral Health-Related Quality of Life (OHRQoL) are important areas of research, with studies showing both positive and negative impacts. Therefore, it was surprising that very few trials (0.9%) were found on this topic. Patient centered research should be highlighted in future research.

Furthermore, health economics (1.1%) and implementation studies (1.1%) were sparingly represented which also was unexpected.

### Strengths and limitations

One of the strengths of our systematic review was that it covered a very long, 55-year timeframe. Another strength was that four examiners with long research experience in RCTs and in the field of orthodontics carried out the assessments. In addition, the inter-examiner agreement was very good. If there were any ambiguities or possible questions, these were resolved by the consensus of the four examiners. Moreover, the search strategy was supervised by a qualified university librarian and the protocol used was a validated tool (RoB2). On the other hand, a limitation of RoB2 was its complexity, and it requires trained individuals to use the tool. Furthermore, using RoB2 is time-consuming to assess [15]. Another limitation was that we used three databases, namely the MEDLINE via Entrez PubMed, Web of Science, and Cochrane library, more databases could have been used.

### Final remarks

We would like to emphasize that our goal with this review of bias in orthodontic RCTs has not been to criticize individual studies with high bias or journals that have published many RCTs with high bias. Rather, the aim has been to demonstrate and raise awareness of the shortcomings in the trials to contribute in the future to establishing a generally improved level of quality in orthodontic RCTs. From this perspective, it is encouraging that the number of RCTs with low bias has increased significantly in recent years and this also applies to publications in the journals that account for the majority of orthodontic RCTs. No one benefits from conducting and producing poor RCTs. Suboptimal methodology and bias, incomplete or incorrect or selective reporting of evidence can greatly lead to ineffective or even potentially harmful treatment interventions [5].

We recommend that researchers and research groups familiarize themselves with RoB2 before drawing up research protocols and research plans. This approach allows them to become familiar with the six domains of bias that can affect the results of an RCT. One effective method is to use the ‘signaling questions of RoB2’ to aid in designing the plans and protocols for the trial's conduct and course. It is also recommended that journal reviewers and editors consider the six domains of bias when assessing submitted research manuscripts. The goal must be for all parties, i.e. researchers, reviewers and editors, to publish as many RCTs with low bias as possible.

## Conclusions

This study clearly shows that improvements in the quality of RCTs in orthodontics are still needed. To diminish the risk of bias, we recommend that researchers and research groups familiarize themselves with RoB2 before drawing up research protocols and research plans. Specifically, they should consider using the ‘signaling questions of RoB2’ as much as possible for help to design the plans and protocols about the trial's conduct and course.

## Data Availability

Available upon request.
